# Change and Removal

**Published:** 1871-10

**Authors:** 


### CHANGE AND REMOVAL.

All our readers know that about three years ago, their and our especial friend, and one of the editors of this Journal, Dr. Geo. Watt, was compelled by ill health to give up for the most part he practice of our profession; but in doing so he could not remain idle, but engaged in the invention, improvement, manufacture and sale of Dental instruments and materials; in these directions he accomplished much for the profession. During this time he also held the chair of Chemistry in the Ohio Dental College, in which he labored most efficiently and acceptably. Within the last few months circumstances made another, or rather other changes necessary. Soon after the close of the last session of the Dental College he regarded his resignation from active labor in that Institution a matter of necessity, his health and strength being such as to absolutely forbid his longer retaining his position. After much hesitancy on the part of the Trustees, and greatly to the regret of all concerned, his resignation was accepted. About the first of September he deemed it best to make still further changes, which resulted in the sale of the Dental Depot, and his removal to Xenia, the place of his former home. He has always regarded Xenia as his home, though absent from it for a time. There is perhaps to each of us some place so dear and sacred in its associations that we can not and do not wish to forget it as home.

There he will engage in the practice of the profession again, as formerly. This removal of Dr. Watt from our city is a matter of great regret to many of his friends, and especially to the profession here. Of one thing we think the profession may rest assured, that so long as health and strength remain, his talents will be exercised for the best interest of a profession he loves so well. May the brightest prosperity attend him in all his course of life. T.

